# Supplementary figures and images for: Microbial biogeography of the wombat gastrointestinal tract
Source: PeerJ. 2022 Feb 23;10:e12982. doi: 10.7717/peerj.12982 (PMC8881912; doi:10.7717/peerj.12982)

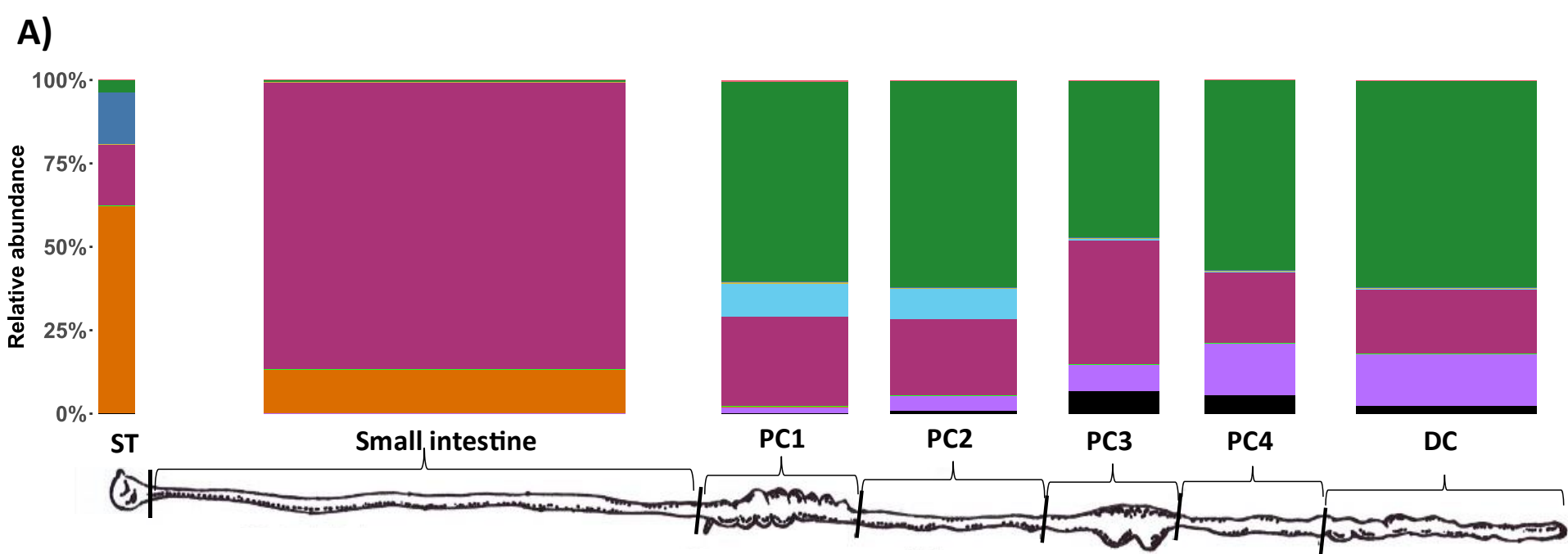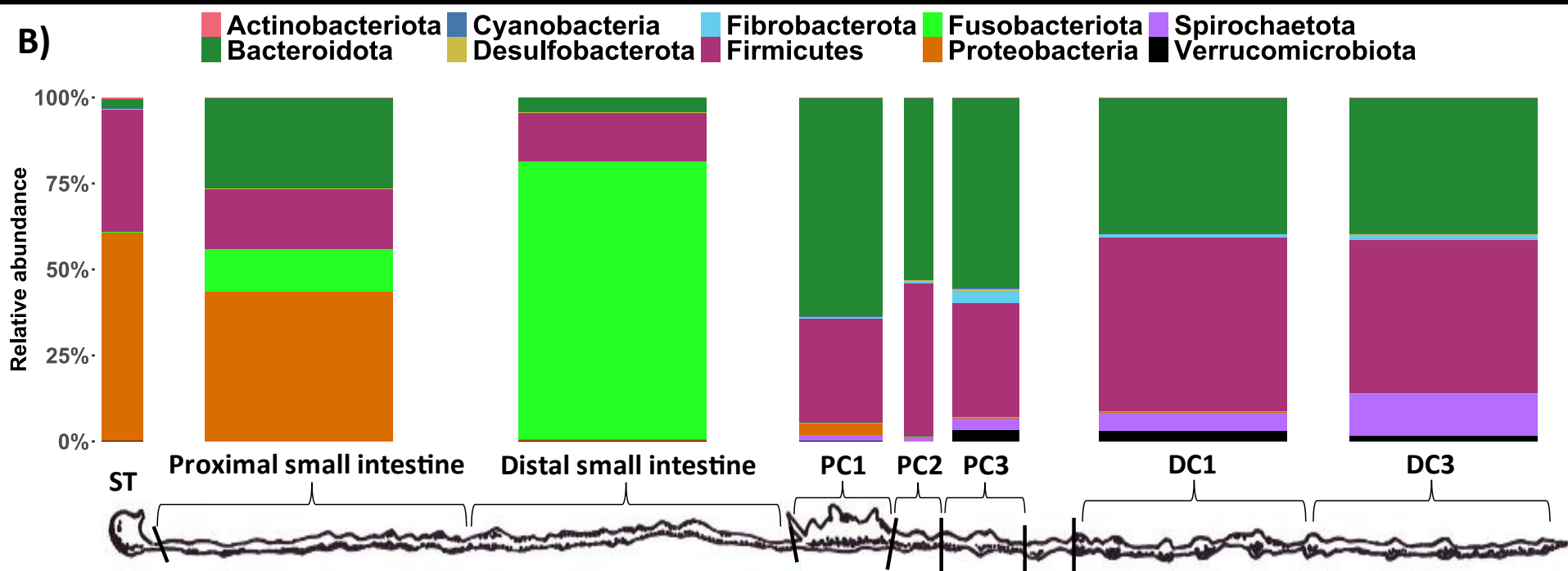

Supplement: Figure S1 — Taxonomic composition of the different sample types at the phylum level. Only the top 10 most abundant phyla are displayed for clarity (these phyla account for >99% of reads). Replicate samples were merged per sample site. (A) bare-nosed wombat. (B) southern hairy-nosed wombat. The widths of the bars are scaled to the length of the GI region. [file peerj-10-12982-s001.pdf]

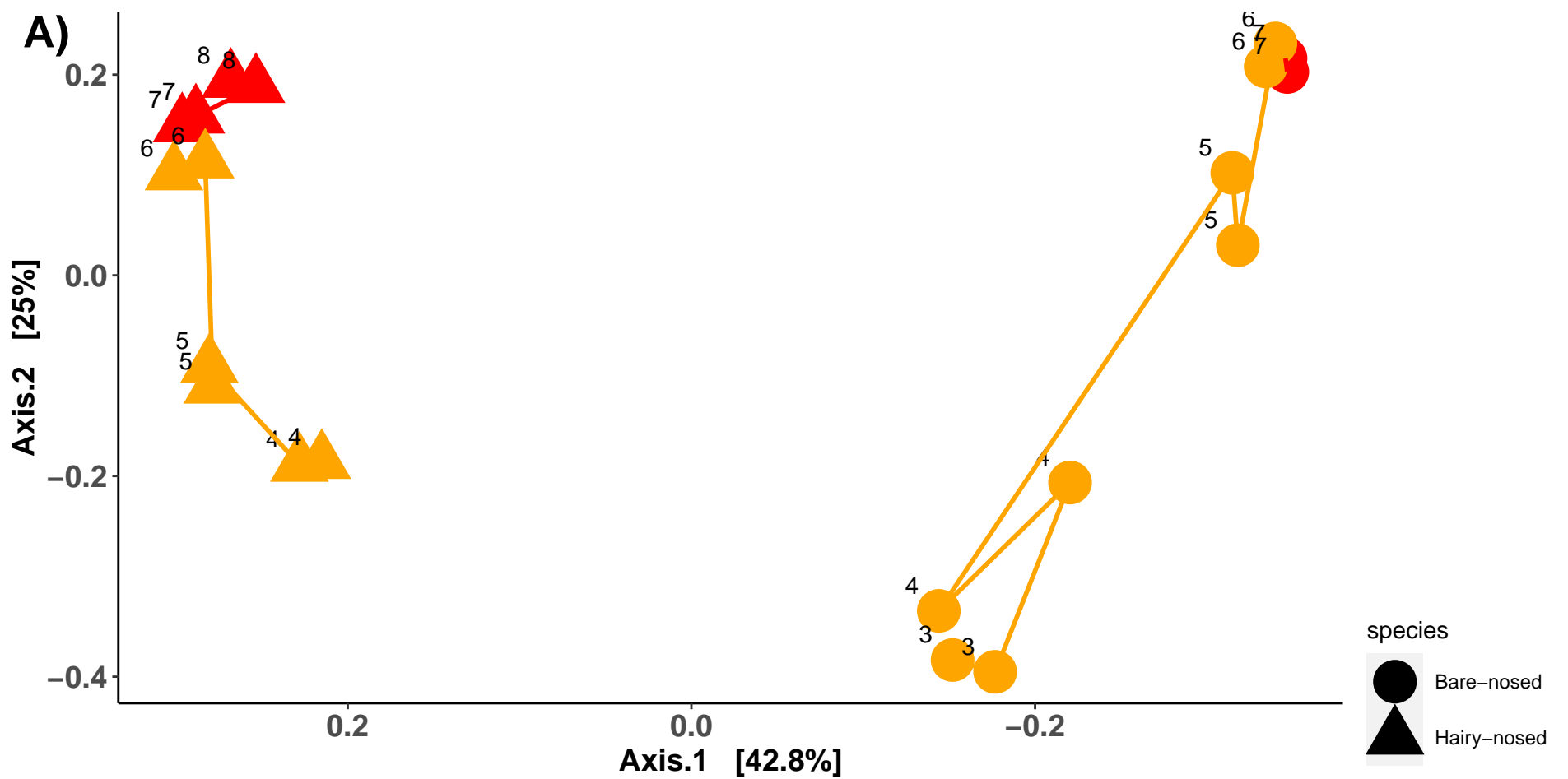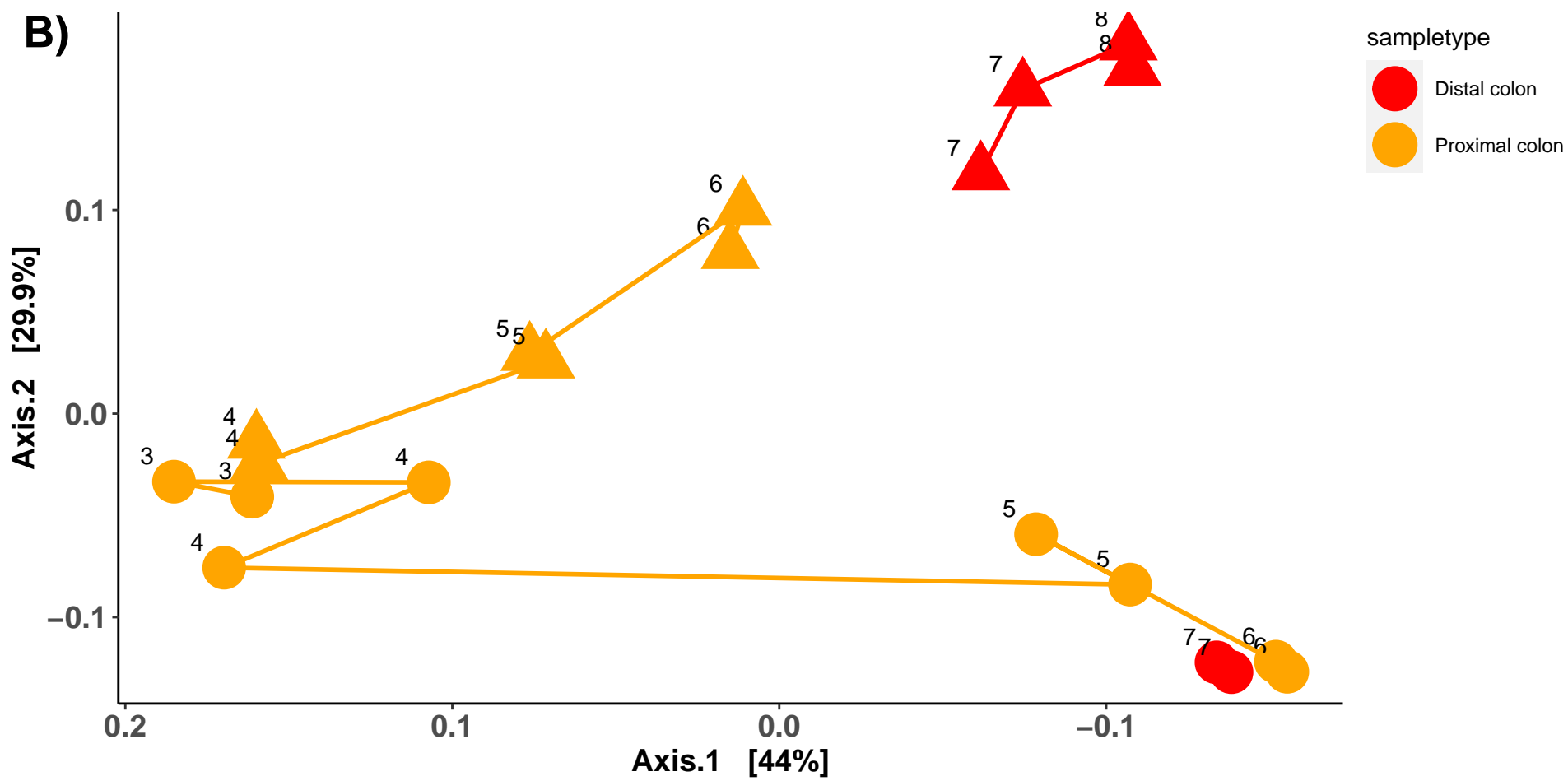

Supplement: Figure S2 — Ordination of colon samples only. (A) PCoA of unweighted UniFrac distances and (B) PCoA of weighted UniFrac distances. Samples are coloured by sample type, and shaped by host species. [file peerj-10-12982-s002.pdf]

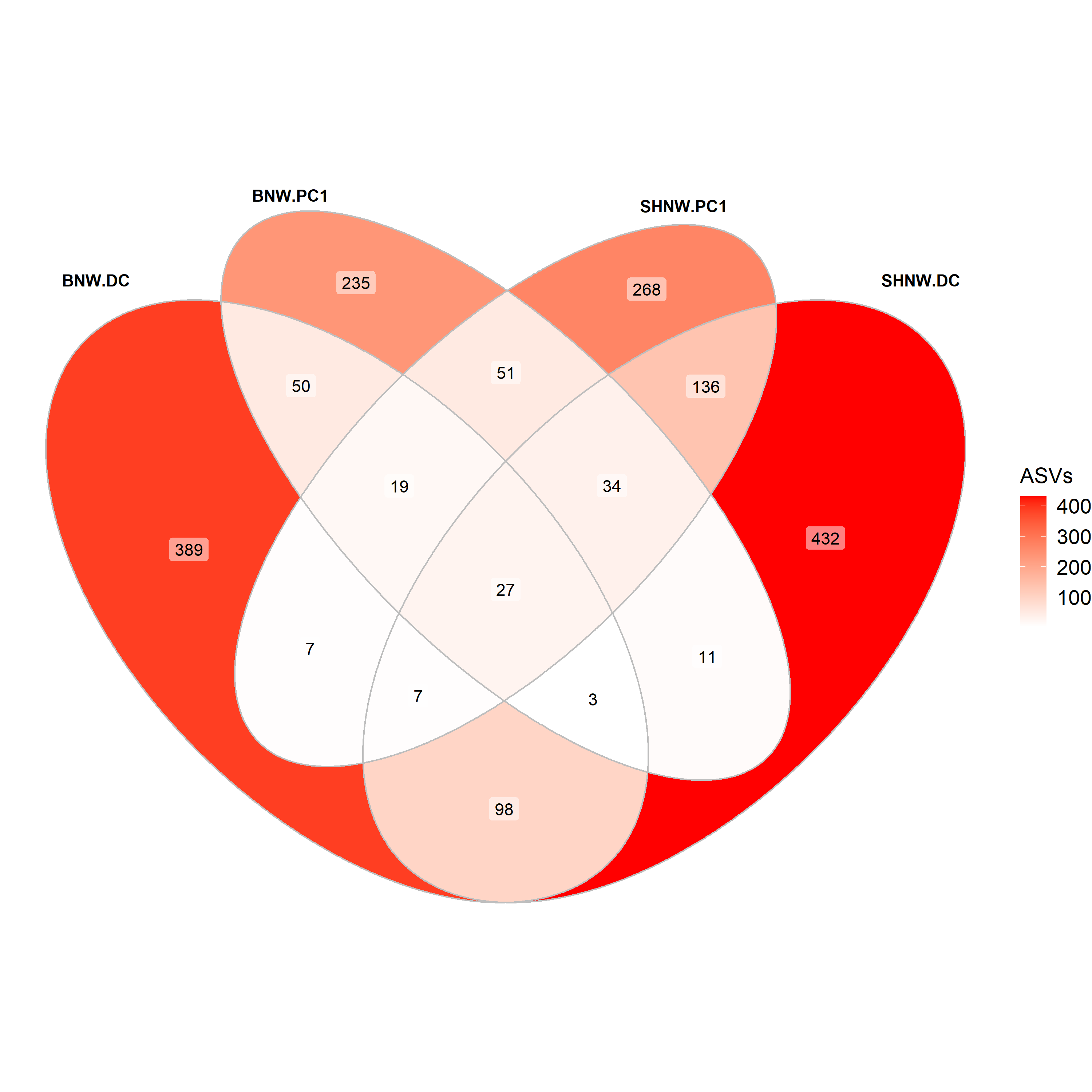

Supplement: Figure S3 [file peerj-10-12982-s003.png]

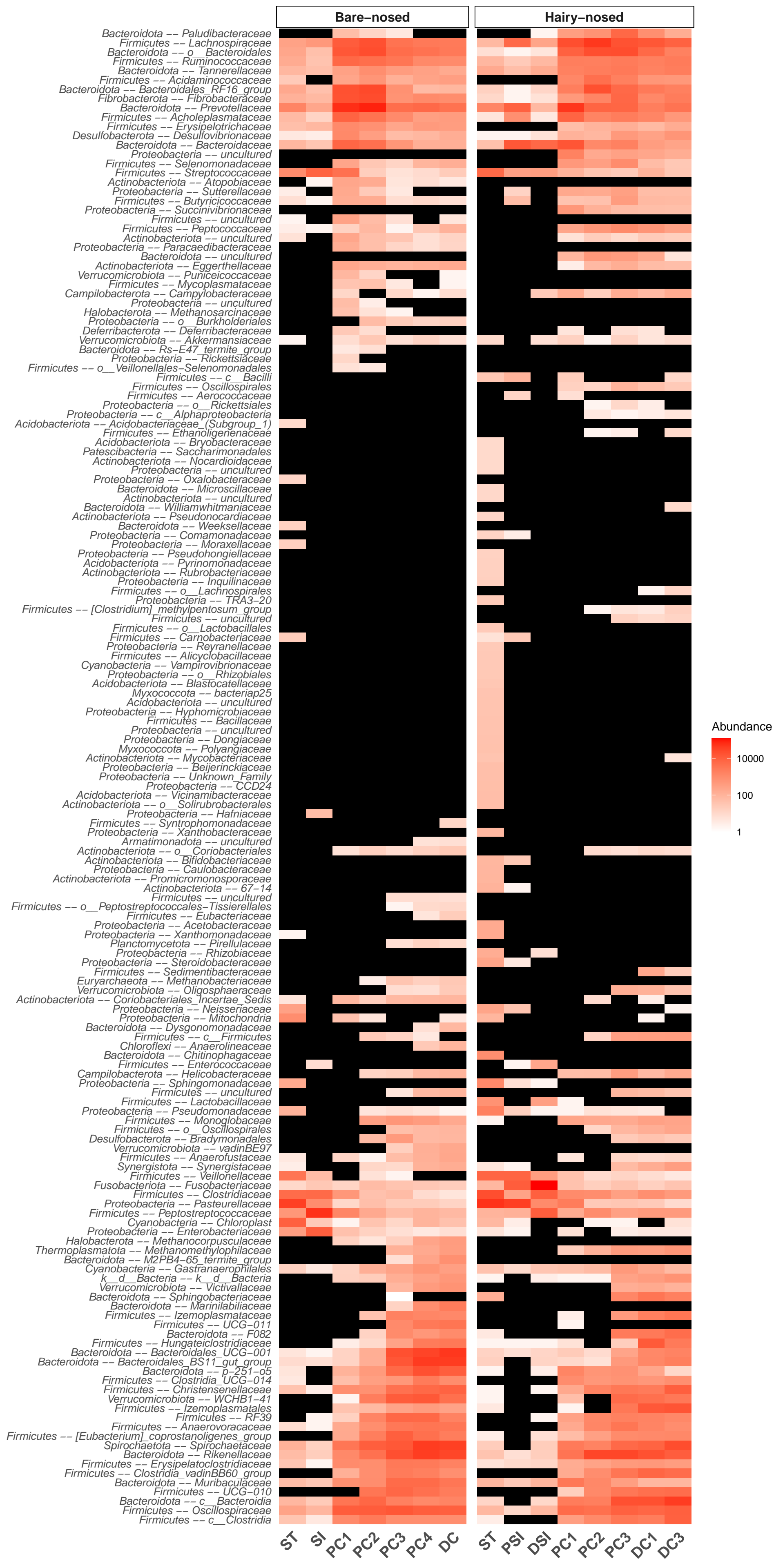

Supplement: Figure S4 — Black indicates 0 assigned reads. Non-family assignments are prefixed with the lowest level of taxonomy that could be assigned (e.g. o– = family). Taxonomy string are prefixed with ‘Phylum –’. [file peerj-10-12982-s004.pdf]

**A)**

**Bare-nosed Wombat**

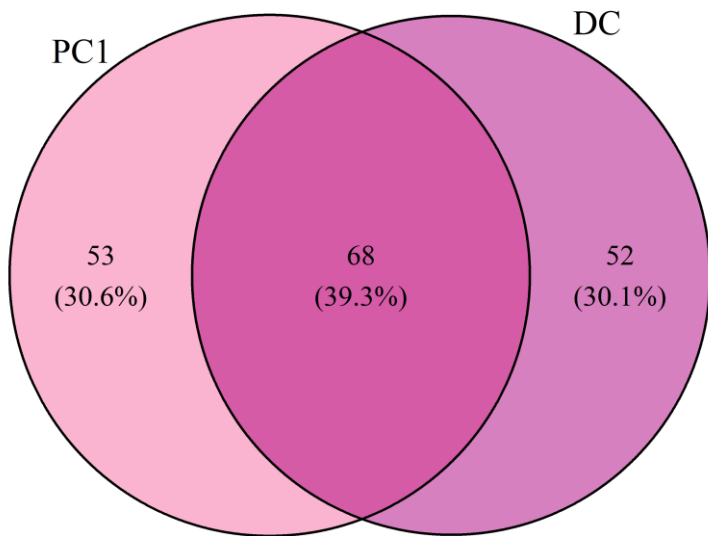

**B)**

**Southern Hairy-nosed Wombat**

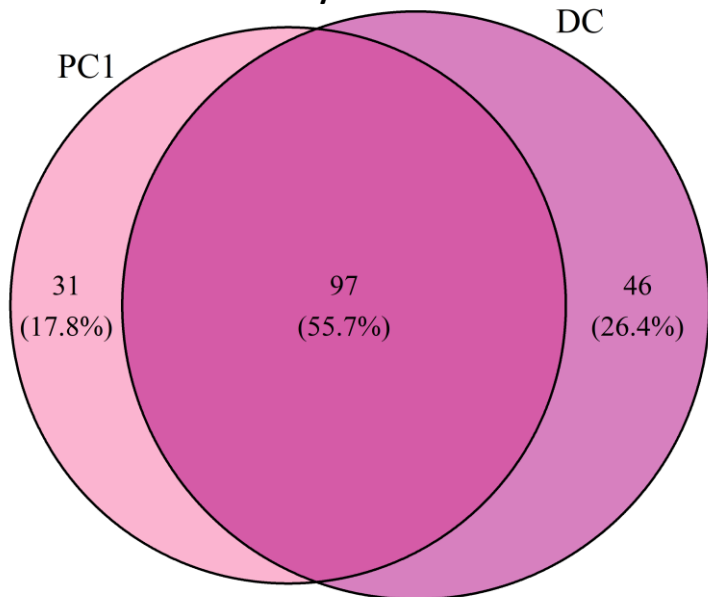

Supplement: Figure S5 — Euler diagram of genera shared between proximal colon 1 (PC1) and last distal colon site (DC) for (A) bare-nosed wombat (BNW) and (B) southern hairy-nosed wombat (SHNW). Percentages represent the proportion of genera specific to a given area. [file peerj-10-12982-s005.pdf]
